# Optimizing Flux Capacity of Dead-end Filtration Membranes by Controlling Flow with Pulse Width Modulated Periodic Backflush

**DOI:** 10.1038/s41598-020-57649-9

**Published:** 2020-01-21

**Authors:** Aaron C. Enten, Matthew P. I. Leipner, Michael C. Bellavia, Lillian E. King, Todd A. Sulchek

**Affiliations:** 1 Georgia Institute of Technology, BioEngineering, Atlanta, 30318 USA; 2 Georgia Institute of Technology, Electrical and Computer Engineering, Atlanta, 30318 USA; 3 Georgia Institute of Technology, Chemical and Biomolecular Engineering, Atlanta, 30318 USA; 4 Georgia Institute of Technology, Biomedical Engineering, Atlanta, 30318 USA; 5 Georgia Institute of Technology, Mechanical Engineering, Atlanta, 30318 USA

**Keywords:** Isolation, separation and purification, Biomedical engineering, Computational science

## Abstract

Standard dead-end sample filtration is used to improve sample purity, but is limited as particle build-up fouls the filter, leading to reduced recovery. The fouling layer can be periodically cleared with backflush algorithms applied through a customized fluidic actuator using variable duty cycles, significantly improving particulate recovery percentage. We show a Pulse Width Modulation (PWM) process can periodically backflush the filter membrane to repeatedly interrupt cake formation and reintegrate the fouling layer into the sample, improving net permeate flux per unit volume of sample by partially restoring filter flux capacity. PWM flow for 2.19 um (targeted) and 7.32 um (untargeted) polystyrene microbeads produced 18-fold higher permeate concentration, higher recovery up to 68.5%, and an 8-fold enrichment increase, compared to a uniform flow. As the duty cycle approaches 50%, the recovery percentage monotonically increases after a critical threshold. Further, we developed and validated a mathematical model to determine that fast, small-volume backflush pulses near 50% duty cycle yield higher recovery by decreasing fouling associated with the cake layer. Optimized PWM flow was then used to purify custom particles for immune activation, achieving 3-fold higher recovery percentage and providing a new route to improve purification yields for diagnostic and cellular applications.

## Introduction

Dead-end filtration using patterned microsieves, fiber meshwork, and membranes of various materials is a standard technique to isolate desired particles of various sizes and is often used in clinical and laboratory settings for therapeutic and diagnostic applications^1–4^. Both biological and physical suspensions can be filtered to yield high purity and enrichment at a high throughput. Dead-end filters are especially susceptible to fouling, however, which leads to lower recovery percentage and yield as a direct result^2,5,6^. Because a decreasing yield negatively impacts therapeutic quality^7–9^, clinical and industrial therapeutic manufacturing will frequently change or increase the surface area of the dead-end filter^10^, or switch to crossflow filtration modalities^11,12^, which further decreases the throughput and increases processing time.

Membrane fouling is caused by pore blocking followed by cake layer formation, resulting in an exponential decay with time in the flux of permeating particulate^13–19^. Membrane fouling also affects crossflow systems and, in this case, numerous studies were conducted to disrupt cake formation and reintegrate particulates into the bulk flow feed stream^11,12,20–25^. For example, crossflow filtration can disrupt caking by implementing an oscillatory flow with a sinusoidal flow velocity or a pulsatile flow, consisting of a steady flow with oscillations superimposed^20^. These studies examined the effects of numerous waveforms, including variations of sinusoids, saw tooth, and square waves^12,20,23^. Oscillatory and pulsatile techniques showed improved clearance of the crossflow membranes, leading to an increase by up to an order of magnitude of flux of permeate across the membrane. There is now a consensus in crossflow systems that reversals in transmembrane pressure (TMP) for short periods of time disrupt accumulated particulates and reduce membrane resistance, improving permeate flux for the processed samples^12,23,26^. However, crossflow filtration suffers from extraordinarily low throughput and requires recirculation of processed samples to achieve higher recovery percentages, straining cellular samples and impacting their morphology and function. More studies have been conducted to analyze the effects of periodic backflush on crossflow than dead-end filtration systems. This may be because dead-end systems lack shear flow removal of cake fouling. Because dead-end filters are ubiquitous in clinical and laboratory settings, solving the fouling problem for this type of filter will be impactful.

**Figure 1:**
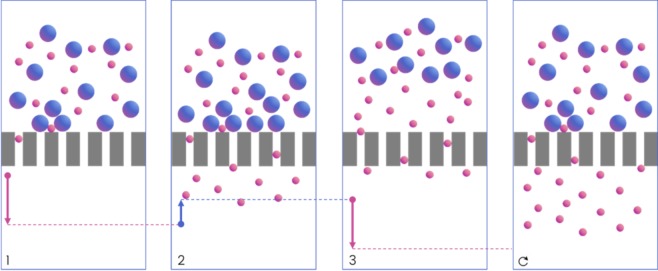
Microfiltration model with periodic backflush. Arrows indicate fluid velocity direction and duration in each step leading the result depicted in the subsequent step. Step (1) forward flush resulting in cake formation and fouling as pore blocking occurs. Step (2) initiation of backflush to remove particle buildup from the cake layer and membrane surface. Step (3) re-initiation of forward flush after backflush has cleared the fouling. Negative flux of permeate particulate occurs during step two and reaches a maximum at start of step three. This is repeated periodically in this order to improve flux and increase permeation and recovery percentage of processed samples.

**Figure 2:**
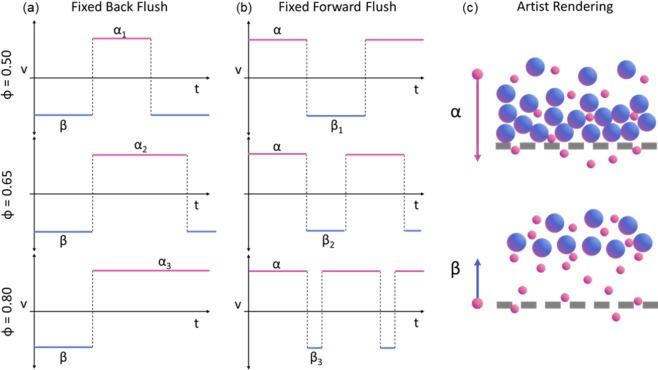
(**a**) Representation of how variations in duty cycle percentage correspond to changes in forward flush duration for fixed back flush. This enables us to hold backflush volume (β), observable in artist rendering, constant while investigating the impact of cake thickness, proportional to forward flush volume (α). (**b**) Representation of how variations in duty cycle percentage correspond to changes in backflush duration. This enables us to hold cake thickness to a consistent value while investigating impact backflush volume. (**c**) Artist rendering of forward and back flush on fouling layer.

Here, we explore how periodic backflush can be applied to dead-end filters and optimized through manipulation of square wave fluid flow through the filter to improve recovery percentage, while maintaining enrichment, purity, and throughput. A schematic of the procedure is shown in Fig. 1. The flow is controlled through pulse width modulation (PWM) of fluidic velocity, characterized by periodicity and duty cycle, Fig. 2. We show the use of PWM backflush for two experimental modalities, Fixed Backflush (FBF) and Fixed Forward Flush (FFF), compared to a uniform forward flow. These experimental modalities allow us to explore the effects of cake thickness on clearance and backflush volume on reintegration respectively. PWM applied to dead-end filtration produced over 18-fold higher permeate concentration, significantly higher recovery percentage of up to 68.5% and increased enrichment of 8-fold. Further, we built a computational model including the interruption of cake formation and reintegration of the fouling layer into the bulk of a sample during PWM periodic backflush, resulting in an improved net permeate flux per unit volume of sample while restoring flux capacity to the filter.

## Results and Discussion

### Fixed backflush

FBF volume flow was conducted to hold backflush constant and independently examine the forward flush durations. From Fig. 2, we know that longer forward flush periods result in larger volumes of bulk solution moved through the membrane for each period. Longer forward flush periods result in increased caking and decrease the ability to clear the fouling due to packing, adsorption, and higher membrane resistance. We show that increased cake thickness should decrease fouling clearance and reintegration, resulting in decreased average permeate flux for a given net volume of processed bulk solution and that we can leverage forward flow times to optimize recovery percentage for particle separation. In order to measure this, we designed fixed backflush volume experiments, described in Fig. 2a, in which we modify the waveform duty cycle while maintaining backflush volume constant.

**Figure 3:**
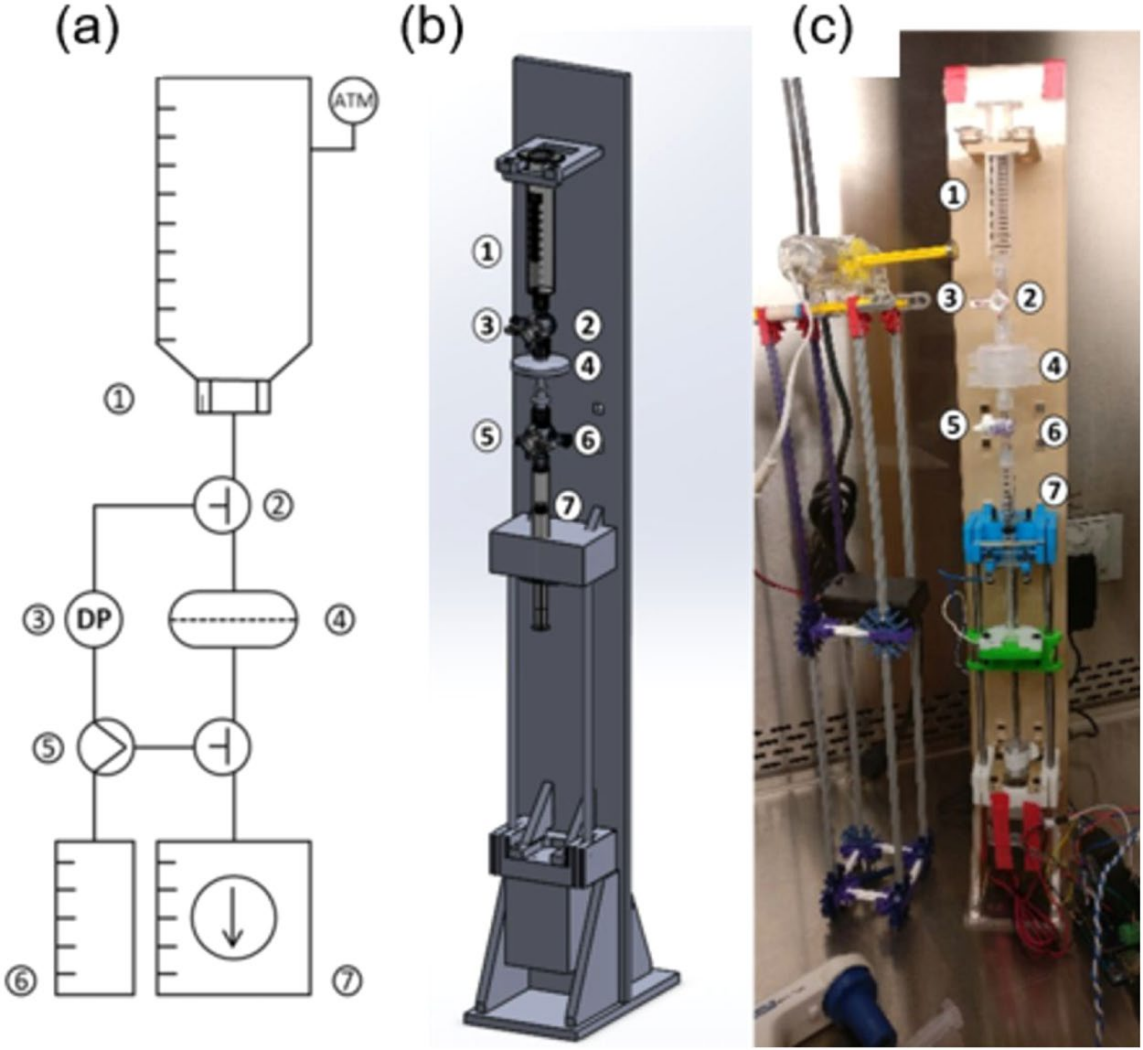
(**a**) Process Flow Diagram, (**b**) 3D rendering, and (**b**) executed PWM programmable syringe actuator prototype [including: 1. Sample reservoir, 2. Four-way stopcock valve, 3. Differential pressure sensor, 4. Dead-end filter, 5. Three-way valve, 6. Priming fluid reservoir, 7. Pressure generating permeate reservoir].

**Figure 4:**
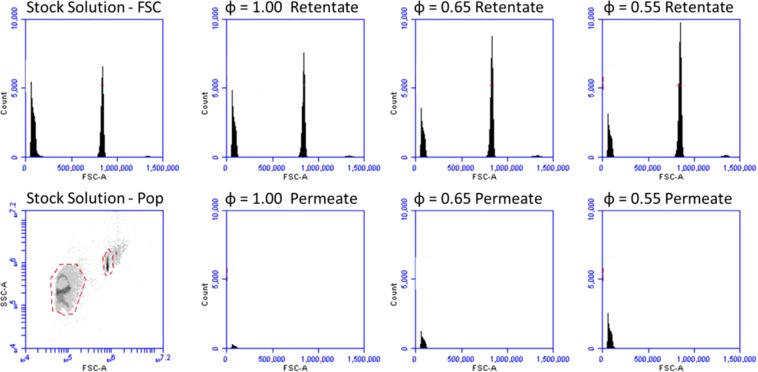
Flow cytometry data showing forward scatter histograms and population dynamics. Images left of dividing line correspond to stock solutions with 2 µm and 7 µm polystyrene microspheres in a 50:50 ratio. Right of dividing line, rows show retentate side and permeate side population dynamics for duty cycles of 1.0, 0.65, and 0.55 respectively in each column. It can be observed that as duty cycle decreases, the 2 µm population quantity decreases in the retentate and increases in the permeate.

The duty cycle of the backflush process was found to impact the target particle concentration at the permeate side of the filter. The filtration system described in Fig. 3 is used to process a mixture of particles. Figure 4 shows the enrichment of smaller 2.19 µm particles from larger 7.32 µm particles initially mixed together for different duty cycles (ϕ). ϕ for this analysis is defined as forward flow volume divided by the gross volume exchange through the filter or the sum of forward flow plus back flow volumes, Eq. (7). In the retentate side row of Fig. 4, the targeted 2.19 µm particle population is shown to decrease as ϕ approaches 0.55. In the permeate side, it is observed that the 2.19 µm particle counts increase significantly without noticeable increase in 7.32 µm particles as ϕ approaches 0.55.

**Figure 5:**
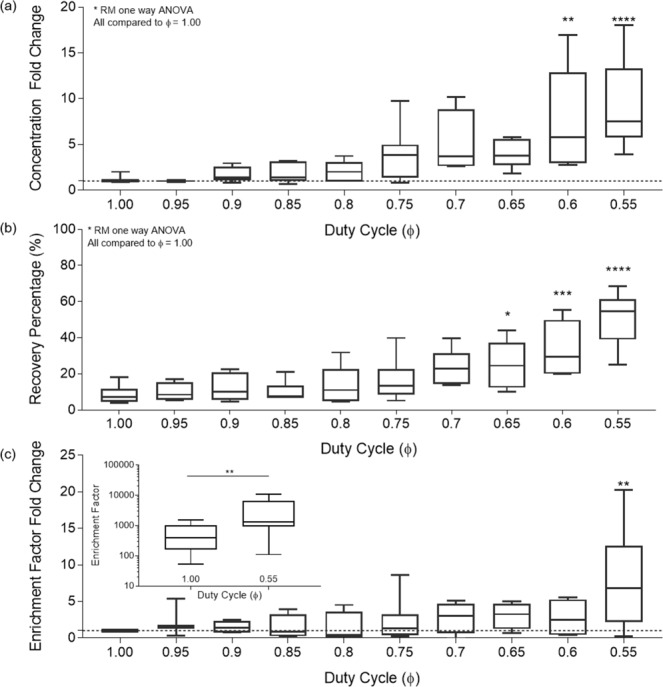
(**a**) Fixed Back Flush concentration fold change as a function of ϕ. Data for n = 10 experiments each were normalized to the concentration at ϕ = 1.0 (Duty cycle percentage 100). Dotted line shows a concentration fold change of 1, corresponding to conventional ϕ = 1.0. (**b**) Fixed Forward Flush Recovery percentage as a function of duty cycle. (**c**) Enrichment Factor Fold Change with respect to duty cycle fraction 1.00. (Subgraph) Enrichment Factor numerical result between ϕ = 1.00 and 0.55.

Particle counts from individual experiments resulting in the images in Fig. 4 were used to calculate a concentration fold change curve. The mean particle concentration between ϕ = 1.0 and 0.95 was calculated, and the concentration data for all ϕ were normalized to this mean to express fold change improvement as a function of ϕ, shown in Fig. 5. We find an average fold change improvement of 7.82 for ϕ = 0.55.

1 $$Recovery\,Percentage=\frac{(target\,particle\,coun{t}_{output})}{(target\,particle\,coun{t}_{input})}\ast 100$$

Recovery percentage, Fig. 5b, shows the number of desired or targeted particles collected at the output compared to the number of desired particles initially supplied at the input, Eq. (1).

The recovery percentage significantly increased (pairwise comparison with t-test p < 0.0001) from a median of 7.11% to 54.73%, for ϕ = 1.0 and 0.55 respectively. Figure 5b shows that recovery percentage follows a bi-linear function with a region of little to no gain from ϕ = 1.0 to 0.8 followed by a region of linear gain from ϕ = 0.8 to 0.55.

Further, microsphere counts were compared to original sample stock to calculate recovery percentage and population percentage changes were used to calculate the enrichment factor^27^ as functions of ϕ. Enrichment factor is a ratio of ratios that shows the proportion of targeted to nontargeted particles in the output compared to the initial sample being processed at the input, Eq. (2). We explore enrichment factor to show that this process does not significantly increase undesired particle counts at the output in conjunction with desired particles.

2 $${\rm{Enrichment}}\,{\rm{Factor}}=\frac{{(\frac{targetted}{nontargeted})}_{output}}{{(\frac{targetted}{nontargeted})}_{intput}}$$

Increasing values of enrichment indicate increasing effectiveness of processing. We observe that increased enrichment factor results from increased recovery percentage of targeted particles at the output with no significant change in the nontargeted output particle counts. Figure 5c shows enrichment significantly improved (p < 0.01) as ϕ approached 0.55, increasing from an average of 567.3 to 3374.8 for ϕ = 1.0 and 0.55, respectively, Fig. 5c.

### Fixed forward flush

**Figure 6:**
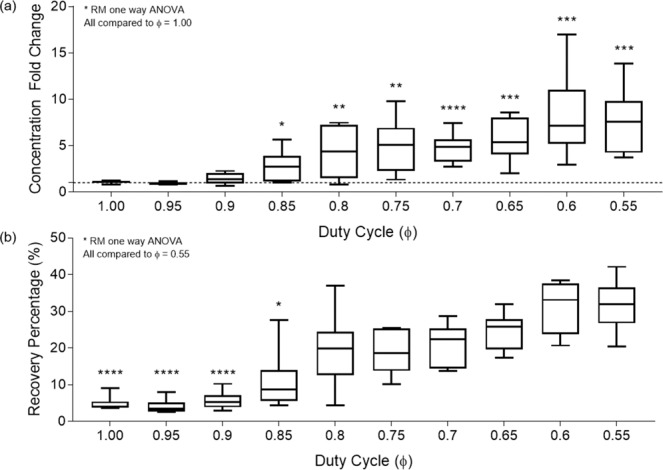
(**a**) Fixed Forward Flush concentration gain as a function of duty cycle percentage from 100 to 55 for n = 10 fixed forward flush volume experiments. (**b**) Fixed Forward Flush Recovery percentage as a function of duty cycle.

The total backflush volume, affecting particle displacement from the filter, can impact breakup and reintegration of the fouling layer. To isolate the impact of backflush volume, we fixed the forward flush volume while varying the backflush volume as a function of ϕ, illustrated in Fig. 2. The total forward flush volume was fixed to a predetermined level to hold cake material buildup consistent in these experiments. The FFF volume was chosen from the FBF volume values which produced minimal but observable changes in concentration. The output concentration increases as the backflush volume increases and these data were normalized as in Fig. 5 to calculate concentration gain, shown in Fig. 6. However, concentration gain saturates at a duty cycle fraction of 0.75 and recovery percentage does not significantly improve beyond this duty cycle value (Fig. 6). Similar to our observations in the FBF results, we observe that smaller periodicities result in improved clearance, but with diminishing net particle flux with larger backflush volumes. We conclude that there exists an optimal recovery percentage that results from the increasing periodicities explored in FBF and the decreasing periodicities of FFF at a duty cycle of 0.55 for the tested backflush volume. Based on this conclusion, we developed a mathematical model to parameterize the results and find optimal enrichment conditions.

### Modelling

In the fixed backflush experiments, we observed a marked change in the permeate output concentrations with decreasing duty cycle, resulting in an improved mean enrichment of target particles of 8.0-fold. Recovery percentage is also critical to improve, and we showed a median value increase from 7.1% to 54.7% as duty cycle varied from 1.0 to 0.55 by implementing PWM flow control. To optimize the recovery percentage of the flow control system, we developed a mathematical model that describes outcomes as a function of duty cycle through periodic cake disruption and re-integration into flow.

We assume a semi-permeable membrane that separate particulates suspended in fluid with a microfiltration process. Filtration through a porous membrane is governed by Darcy’s law, in which permeate flux, *J*, is proportional to the differential pressure drop, *ΔP*, through a given thickness, *dz, of a membrane* as per Eq. (3) ^15^.

3 $$J=\frac{1}{{A}_{M}}\frac{dV\{t\}}{dt}=-\frac{k}{\mu }\frac{\Delta P}{dz}$$

Darcy’s law can be modified assuming a constant pressure drop across the membrane in which the right-hand side of Eq. (3) can be simplified to:

4 $$J=\frac{1}{{A}_{M}}\frac{dV\{t\}}{dt}=\frac{\varDelta P}{\mu ({R}_{m}+{R}_{c})}$$

where R_m_ is the resistance due to the membrane and R_c_, the time-dependent resistance of the filter cake^15^.

A variety of fouling models for R_c_ are used to test whether pore-blockage, pore-constriction, or cake formation dominate at each stage in filtration. A model proposed by Ho and Zydeny^28^ accounts for a transition between R_c_ models for protein fouling at all times through the dead-end filtration process as follows:

5 $${{\rm{R}}}_{{\rm{c}}}=({{\rm{R}}}_{{\rm{M}}}+{{\rm{R}}}_{{\rm{c}},0})\sqrt{1+\frac{{{\rm{f}}^{\prime} {\rm{R}}^{\prime} \Delta \text{PC}}_{{\rm{b}}}}{{\rm{\mu }}{({{\rm{R}}}_{{\rm{M}}}+{{\rm{R}}}_{{\rm{c}},0})}^{2}}({\rm{t}}-{{\rm{t}}}_{{\rm{p}}})}-{{\rm{R}}}_{{\rm{m}}}$$

This relation is then implemented into Eq. (4) yielding Eq. (6).

6 $$J={J}_{0}[\exp (-\frac{\alpha \varDelta P{C}_{b}}{\mu {R}_{m}}t)+{\int }_{0}^{t}\frac{\alpha \varDelta P{C}_{b}}{\mu ({R}_{m}+{R}_{p})}\times \exp (-\frac{\alpha \varDelta P{C}_{b}}{\mu {R}_{m}}{t}_{p})d{t}_{p}]$$

In the case of microfiltration of moderate solute concentration at a significantly larger particle size compared to proteins through a cellulose membrane, we hypothesize that the solvent flux can be assumed constant with time while the solute flux decreases exponentially to zero as t increases. This decrease in solute flux is the primary limitation in dead-end filtration^10,22,29^. We show that implementing a PWM periodic backflush cycle to clear reversible fouling of the membrane from cake and pore under these conditions can increase solute flux, recovery, and enrichment factor. The ratio of forward flush volume *V*_*f*_ to backflush volume *V*_*r*_ during a filtration operation is the duty cycle, ϕ:

7 $${\rm{\phi }}=\frac{{{\rm{V}}}_{{\rm{f}}}}{{{\rm{V}}}_{{\rm{f}}}+{{\rm{V}}}_{{\rm{r}}}}$$

A ϕ of 1, V_f_ = V_f_ + V_r_, constitutes continuous forward flush with no backflush and is subject to standard exponential decay in flux as fouling occurs. Comparatively, ϕ = 0.50, V_f_ = (V_f_ + V_r_)/2, results in the solvent volume dedicated to forward flush through the membrane equal to the solvent volume for backflush in any given periodic cycle and no net processing would occur.

The profile of a microfiltration process with fixed periodic backflush volume is shown in Supplement 3. With backflush volume fixed, increasing duty cycle result in increased total forward flush volume and increasing cake thickness. Therefore, as ϕ increases, the cake thickness should grow proportionally. The forward flush volume is calculated as a function of ϕ and backflush volume per Eq. (8):

8 $${{\rm{v}}}_{{\rm{f}}}={{\rm{v}}}_{{\rm{r}}}\frac{{\rm{\phi }}\,}{1-{\rm{\phi }}}$$

The process associated with forward flush, Step I, results in an exponential decay period. The process associated with negative flux, Step II, results in permeate particulate rescinding across the membrane during backflush. Step III begins with a low resistance forward flux period immediately following backflush in which the back-flushed permeate associated with the negative flux from Step II is pushed through the membrane again, after which, exponential decay of solute flux again takes place. This cycle repeats for the entire volume of the sample processed, Fig. 1. The benefit of this scheme is that the negative flux drastically changes forward flux resistance, resulting in a net improvement in forward flux. In this model, we assume an infinite bulk supply, keeping the exponential decay rate constant for each successive cycle, and incorporated irreversible fouling, resulting in sequential loss of flux capacity after each backflush cycle.

For each cycle, the model assumes a linear relation between duty cycle and the clearance of reversible fouling^30^ per Eq. (9).

9 $${{\rm{R}}}_{{\rm{c}},{\rm{n}}+1}=(1-{\rm{\phi }}){{\rm{R}}}_{{\rm{c}},{\rm{n}}}$$

**Figure 7:**
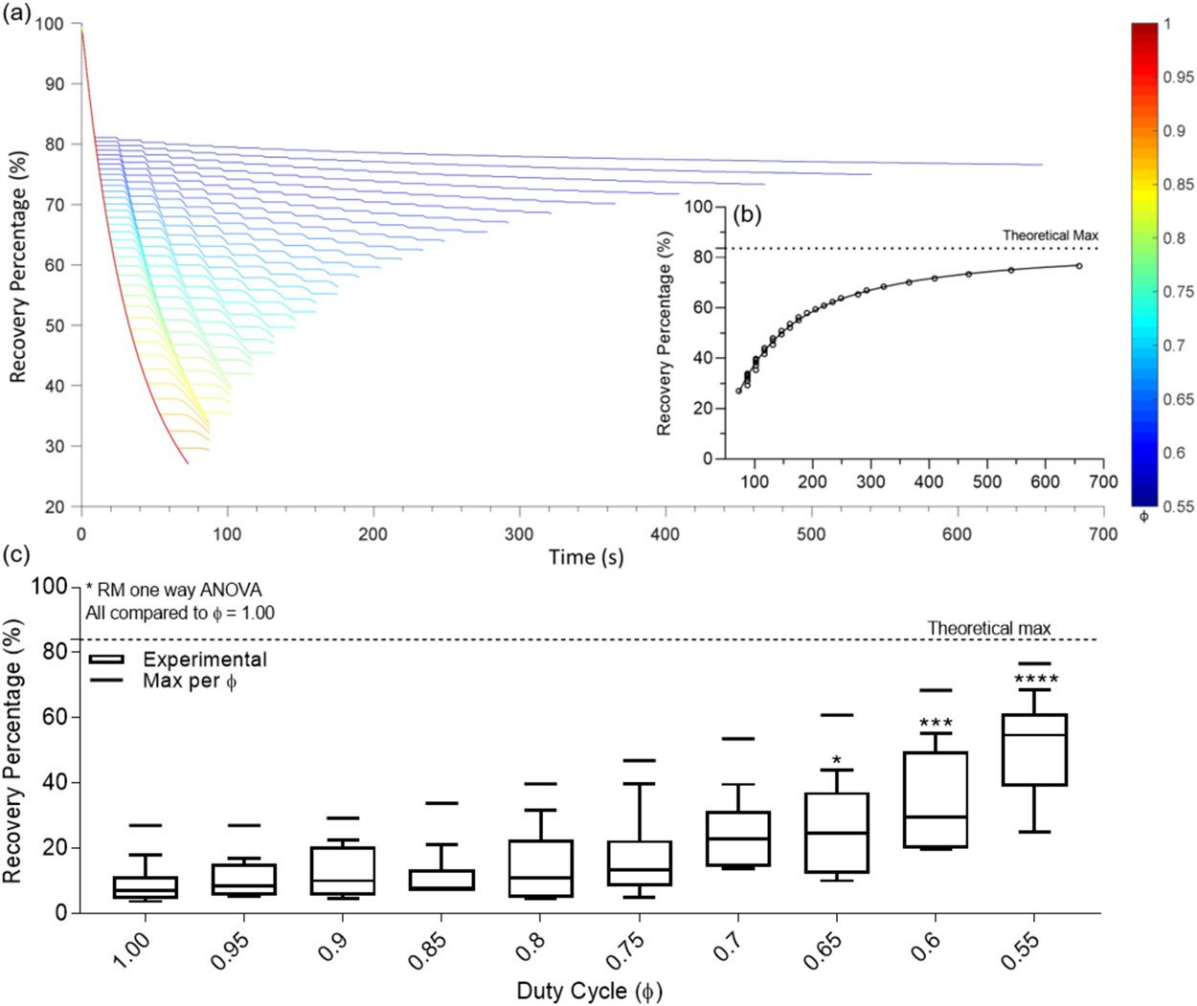
(**a**) Recovery Percentage as a function of time for ϕ from 0.55 to 1.0 in 0.01 increments. (Subgraph) End times for ϕ from 0.55 to 1.0 and calculation for maximum percent recoverable at ϕ = 0.50^+^. (**b**) Theoretical maximum for recovery percentage as a function of time showing asymptotic gain with a maximum at y-asymptote (ϕ = 0.5). (**c**) Fixed Backflush Recovery percentage as a function of duty cycle compared to theoretical maxima at each duty cycle fraction.

As ϕ varies from 1 to 0.50, a greater fraction of the reversible cake fouling is removed. Greater proportional fractions of backflush, i.e. as ϕ approaches 0.50, result in larger resuspensions of solutes from the cake into the bulk, increasing flux capacity for the system, and improving overall permeate recovery. Plotting permeate solute mass and solute flux as functions of time show increased instantaneous slope after backflush, Supplement 3, resulting in monotonically increasing recovery percentage. Figure 7a explores the impact various values of ϕ would have on overall recovery, Eq. (1). Figure 7b shows the increase to a maximum achievable recovery percentage of approximately 84% as ϕ approaches 0.50.

**Figure 8:**
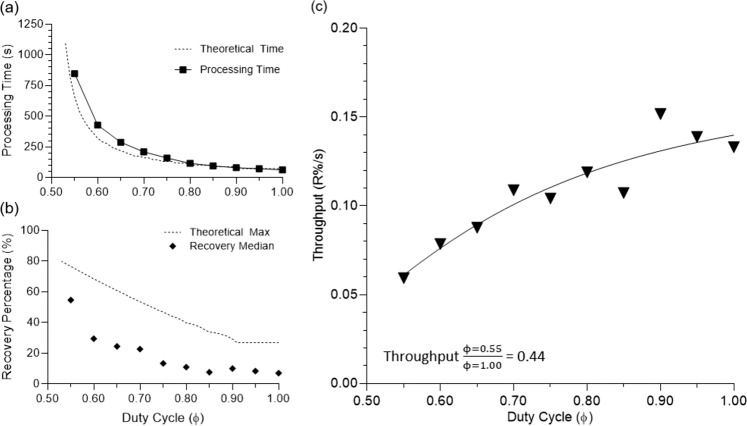
(**a**) Theoretical process time compared to observed process time as a function of ϕ. (**b**) Theoretical maximum for recovery percentage as a function of ϕ compared to median recovery percentage. (**c**) Throughput, calculated as recovery percentage divided by processing time, as a function of ϕ. Reduction of 44% throughput is observed as duty cycle decreases from 1.00 to 0.55.

One consequence of this approach to maximize recovery is illustrated in Fig. 8. As ϕ approaches the minimum value of 0.50, the time to process a sample of bulk concentrate asymptotically approaches infinity. Additionally, we observed a critical duty cycle as ϕ nears 1.00 in which the calculated forward flush volume is greater than the total processed volume, which results in an effective ϕ of 1. Comparing percent recovery versus ϕ to percent recovery versus time, we can show a diminishing improvement of recovery per unit increase in process time, Fig. 8. Further, we can calculate a maximum recovery percentage for each modeled duty cycle to see that the experimentally collected data closely follow the theoretical model and strongly correlates with a Spearman’s correlation coefficient of 0.985. We conclude that from the strong correlation of the model to the experimental data that the separation of the solvent and solute resistance in cake is essential in dead-end particulate separation. Further, we have not observed a model calculated maximum recovery percentage in experimental results, suggesting further room for improvement of our device, Figs. 7c and 8b. The difference between model predictions and experimental data could be due to hysteresis of syringe motion and particle settling and adsorption. The error between theoretical and experimental data could be reduced by increasing syringe precision to minimize spread and improving the model by accounting for experimental losses from settling, adsorption, and human error.

### Applications to improve manufacturing of immune engineering particles

**Figure 9:**
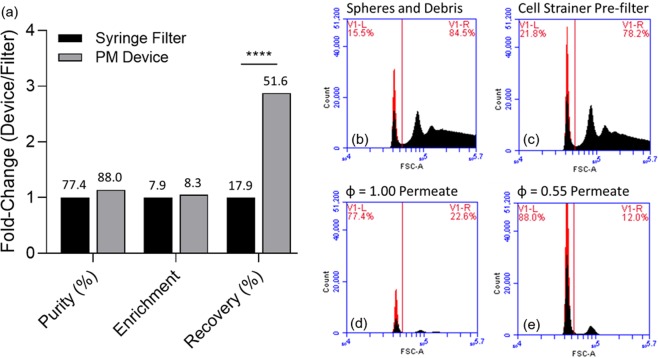
(**a**) Purity, recovery percentage, and EF for conjugated microspheres. ϕ = 1.00 compared to ϕ = 0.55 normalized to ϕ = 1.00 with non-normalized base values provided above bars. B) Conjugated microsphere forward scatter immediately following conjugation, and (**c**) representative population after conjugation and pass through large pore cell strainer. (**d**) Representative population of (**b**) passed through dead-end filter at ϕ = 1.00. High loss is apparent, but target sample purity is high. (**e**) Representative population of (**b**) passed through dead-end filter at ϕ = 0.55. Purity is maintained in a similar proportion to that in (**d**), but population count is, on average, 2.88 times higher.

Filtration of microparticles is frequently used to generate high purity particles for diagnostics and therapeutics and is useful *in-vitro* to study biological phenomena^31,32^. Considering this need, it is significant to demonstrate that we can apply PWM periodic backflush improve recovery of microspheres after protein conjugation protocols. We show periodic backflush can be used to streamline the functionalization process of silica microparticles, 0.96 µm in average diameter, and to improve purity and yield by removing aggregate particulates and debris that results from the functionalization process through filter flux capacity restoration via PWM periodic backflush. Debris was observed to be cleared from the functionalized bead population, Fig. 9, and PWM periodic backflush with ϕ = 0.55 provided purity equivalent to ϕ = 1.00 with significantly higher particle recovery percentage (p < 0.001), Fig. 9. Up to 3-fold greater than what was observed. Further, enrichment did not see significant gain compared to the base case, ϕ = 1.00. Enrichment fold-change enhancement is expected if only targeted particle concentration increases in the output, Eq. (2). As can be seen in Fig. 9d,e, although the quantity of particles per bin increases from an average of 15,000 to over 50,000, the ratio of targeted to untargeted particles in the output remains approximately the same at 77.4% to 22.6% for the case presented at ϕ = 1.00 and 88.0% to 12.0% at ϕ = 0.55.

Experimental investigations into conjugated microsphere separations provide a few critical insights from these experiments. PM backflush is a reliable option for not only separating smaller particles than initially tested, 0.96 µm compared to 2.19, but also those made from various materials, silica compared to polystyrene. Additionally, we show from these experiments that we can apply PWM to improve yield from uniformly distributed debris fields containing multiple irregularly shaped materials.

In this case, we expect reduced recovery percentage and enrichment compared to the polystyrene particle experiments. This reduction can be attributed to the debris field content and particle functionalization. Functionalization would increase both specific and nonspecific binding to other particulate and filter media. These factors increase the density and irreversibility of caking and lead to poorer outcomes. We show that filtration recovery percentage can be improved for functionalized particles without interfering with their effectiveness or losing significant quantities through prolonged contact with membranes potentially increasing losses via adsorption. Finally, we show a median of approximately 3x fold change in recovery percentage, increasing yield, while maintaining purity and enrichment.

## Conclusion

We investigate the effects of pulse width modulated periodic backflush with variable duty cycle on cake formation interruption, fouling layer reintegration, and permeate flux improvement. Utilizing backflush volume, forward flush volume, and frequency, we showed that PWM backflush cyclically restores flux capacity of dead-end filters. We developed a mathematical model to demonstrate that variation in the PWM duty cycle direct impacts recovery percentage and enrichment factor in dead-end systems, and that, experimentally, PWM backflush produces up to 18 times higher permeate concentration, significantly higher (p < 0.05) recovery percentages of 54.7% median compared to a baseline median of 7.1%, and significantly larger (p < 0.01) enrichment factor with an average fold change of 8.0, compared to constant flow rate. Further, we applied this to antibody functionalized polystyrene microspheres to show that we can significantly improve yield for common particle-based reagents with the intention of decreasing cost for these applications.

## Methods

### Membranes

The experiments were conducted with Pall Acrodisc syringe filters with Versapor (hydrophilic polypropylene). The filters are commonly used for both aqueous and organic samples with a glass-fiber prefiltration component. All filters have a diameter of 25 mm and functional cross-sectional area of approximately 2.8 cm2.

### Flow control system

A custom syringe actuator, Fig. 3, was constructed to inject preprogrammed PWM pressure waveforms. The filtration system, composed of a linear actuator, syringe holster, TMP measurement sensor, and magnetic mixer, is mounted vertically. The feed reservoir is open to atmospheric pressure, and the linear actuator manipulates flow by alternating the pressure driven flow movement direction from the permeate side outlet reservoir, Fig. 3. A custom user interface designed in LabVIEW, communicates with an Arduino Uno R3 with custom motor shield to control a stepper motor which in turn translates rotational motion to linear actuation of a syringe. The fluid velocity, pulse duration, duty cycle (ϕ), and frequency of the flow cycle are controllable through the LabVIEW interface. In this system, because flow rate will be held constant for all experiments, periodicity is representative of the gross volume exchange across the filter within a given cycle. Duty cycle, on the other hand, is representative of the ratio of forward flush, V_f_, to backflush, V_r_, and is proportional to net volumetric flux per cycle.

Process parameters are explored by holding the volume exchanged in either the backflush or forward flush components of the duty cycle constant and varying ϕ. By holding the backflush volume or beta component of the duty cycle constant, variations in ϕ result in a changing periodicity that enables us to draw conclusions about the effects of cake layer buildup on reintegration for a given backflush volume. Also, by holding forward flush volume constant and varying ϕ, we can draw conclusions on how backflush volume affects cake disturbance and reintegration for a given cake deposition rate.

The actuator executes syringe displacements in discrete increments with a minimum resolution of 0.23 µL when using a 3-mL BD syringe. The software records inputs of actuator speed, duty cycle percentage, gross volume exchange, and total volume of sample to process. All experiments were conducted at a positive and negative absolute flow rate of 4.55 mL/min, which was found to minimize pressure fluctuations determined through testing with a differential pressure sensor. The system utilizes a reservoir for holding up to 10 mL of sample open to atmospheric pressure, can integrate any Luer-lock based commercially available syringe filters, and measures transmembrane pressure at a sample rate of 50 Hz.

### Waveforms

The system contains an integrated differential pressure, MPXV7025DP-ND, sensor to directly measure changes in TMP ± 3.63 PSI, Fig. 3. This measurement directly correlates to changes in total membrane resistance (R) as a function of cake layer deposition (R_f_).

10 $$TMP={J}_{T}\ast ({R}_{m}+{R}_{f})\ast {\mu }_{T}$$

where:

J_T_ = total solute flux across the membrane

µ_T_ = fluid viscosity

For both fixed backflush and fixed forward flush volume experiments, square wave based actuation of a 3-mL syringe were used to create a square fluid velocity profile measured indirectly through TMP with negative backflush pressures compared to baseline. Duty cycle was varied from 0.55 to 1.00 in increments of 0.05. An example of TMP wave profiles is provided in the supplement for a DI sample at duty cycles of 1.00 and 0.75. This characterization waveform is descriptive of fluid behavior in the system.

### Particulate sample processing

Mixtures of polystyrene (PS) microspheres were suspended in phosphate buffered saline solution with 0.02% tween by volume in PBS, and were transferred to the system for processing via syringe. The particles included mixtures of 2.19 µm and 7.32 µm average diameter polystyrene particles (Bangs Labs) in a 50:50 ratio at a concentration of five million particles/mL, Fig. 4. Particles were flowed through the filter using a syringe pump. Fixed backflush and fixed forward flush volume experiments used to process 1 mL of sample fluid at an absolute flow rate of 4.55 mL/min. For each experiment, the testing rig is primed with DI water prior to testing to eliminate bubbles and reduce capacitive actuation effects.

Additionally, silica microparticles, 0.96 µm in diameter (Bangs Laboratories, Fishers, IN), were coated in 5 nm chromium and 20 nm overlaying gold for functionalization as described by Tang and Holt, *et al*.^33,34^. The particles were filtered in fixed backflush experiments to confirm that beads designed for immunoengineering experiments could be efficiently purified^35^. Prior to experimentation these suspensions were washed and suspended in DI water by centrifugation (3500 g for 15 minutes) to remove buffers and preservatives. Functionalized particles were used in volumes up to 10 mL to demonstrate applicability of PWM periodic backflush within laboratory particle processing applications.

### Volumetric normalization

After processing the total volume of microsphere suspension samples through the membrane using a PWM technique, all samples were normalized for volumetric differences in the priming stage arising from minor variations in the negative space of the stopcocks and filters using the following procedure. The output from both permeate and retentate were collected in 5 mL culture tubes and centrifuged at 3500 g for 15 minutes. The supernatant was removed, and the particle pellets were resuspended in 3.4 mL of DI water. All samples were then vigorously vortexed and kept on a tube rotator to break up the pellet and ensure uniform dispersion through the solution. The particle count for each condition was then measured using flow cytometry.

## Supplementary information

Supplementary Information.

## Data Availability

We will make requested data and associated protocols available to readers without undue qualifications in material transfer agreements upon request through the corresponding author as appropriate. All materials are commercially available through third party distributors.
