# Supplementary material for: Optimizing Flux Capacity of Dead-end Filtration Membranes by Controlling Flow with Pulse Width Modulated Periodic Backflush
Source: Sci Rep. 2020 Jan 21;10:896. doi: 10.1038/s41598-020-57649-9 (PMC6972749; doi:10.1038/s41598-020-57649-9)
Supplement: Supplementary file 1 — Supplementary Information. [file 41598_2020_57649_MOESM1_ESM.pdf]

Optimizing Flux Capacity of Dead-end Filtration Membranes by Controlling Flow with Pulse Width Modulated Periodic Backflush

Aaron Enten[1,*], Matthew Leipner[2], Michael Bellavia[3], Lillian King[2], Todd Sulchek[4]

[1]Georgia Institute of Technology, BioEngineering, Atlanta, 30318, USA
[2]Georgia Institute of Technology, Chemical and Biomolecular Engineering, Atlanta, 30318, USA
[3]Georgia Institute of Technology, Biomedical Engineering, Atlanta, 30318, USA
[4]Georgia Institute of Technology, Mechanical Engineering, Atlanta, 30318, USA
*Corresponding Author

Contact for Corresponding Author
Address: 315 Ferst Drive NW, Atlanta, Ga 30318
Email: todd.sulchek@me.gatech.edu

Supplementary Figure S1: Pressure waveform profile showing transmembrane pressure for duty cycles ϕ = 1.0 and 0.75 for square wave fluid velocity input

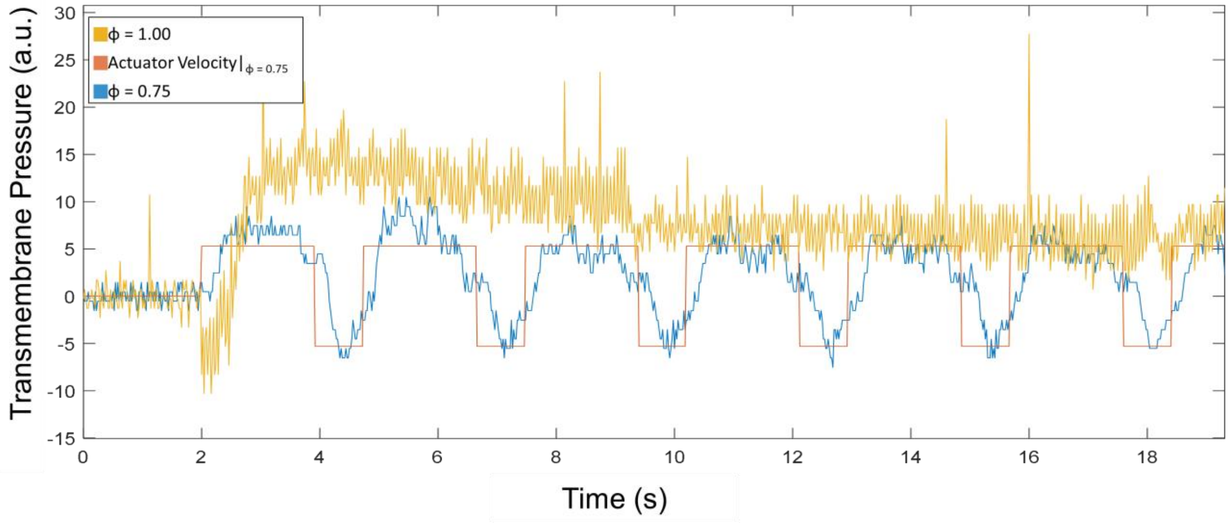

Supplementary Figure S2: Microsphere suspension confocal images (scale bar 5 µm) and population distribution flow cytometric results. A) Shows standard mix of microspheres. B) Variance in concentration can result in slightly more dilute or concentrated suspensions. C) and D) Less than three percent of the population consists of particles bound to other particles (indicated by white arrows). This population can be seen in the forward scatter histogram (E) and forward scatter height vs area plot (F).

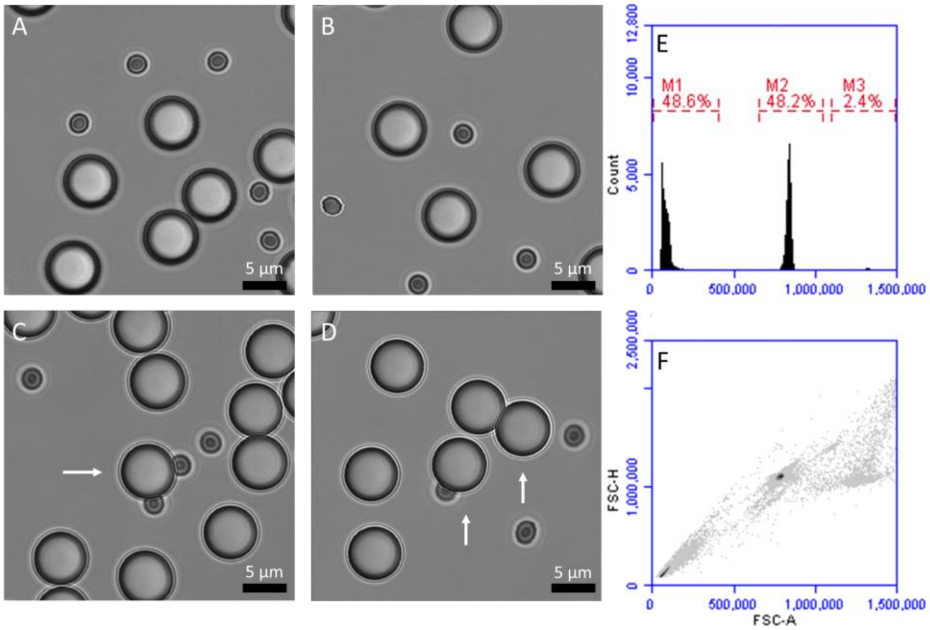

Supplementary Figure S3: Microfiltration model with periodic backflush, ϕ = 0.75. Flux profile in blue, permeate mass in red.

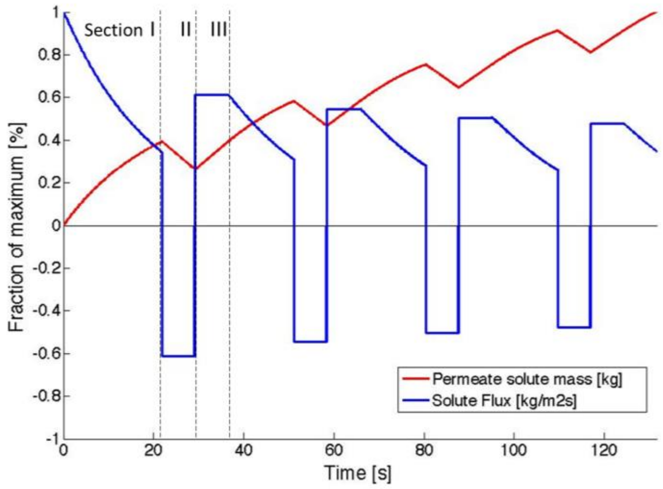

Supplementary Figure S4: Correlation of median experimental to theoretical recovery percentage

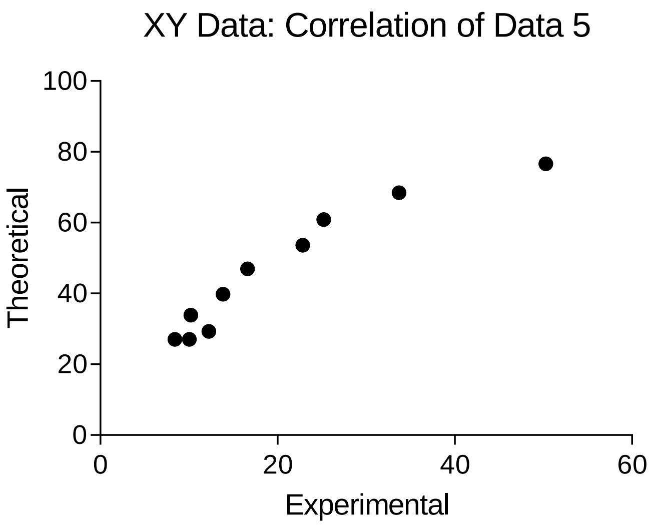

Supplementary Figure S5: Processing time and recovery percentage compared together as functions of duty cycle.

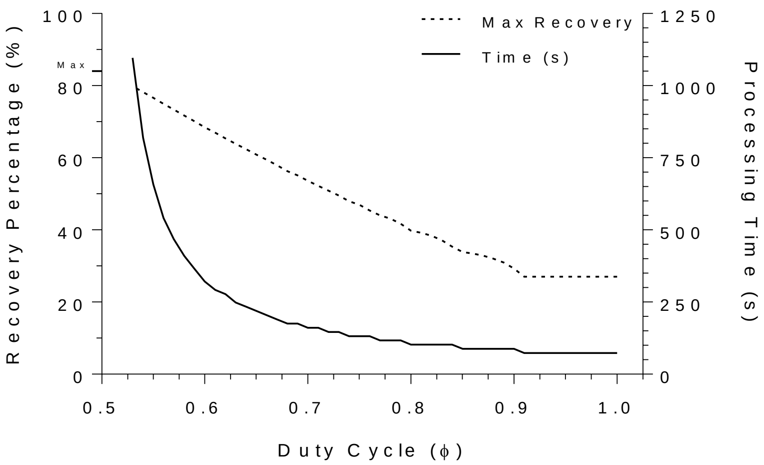

Supplementary Figure S6: Scanning Electron Microscope image of membrane surface

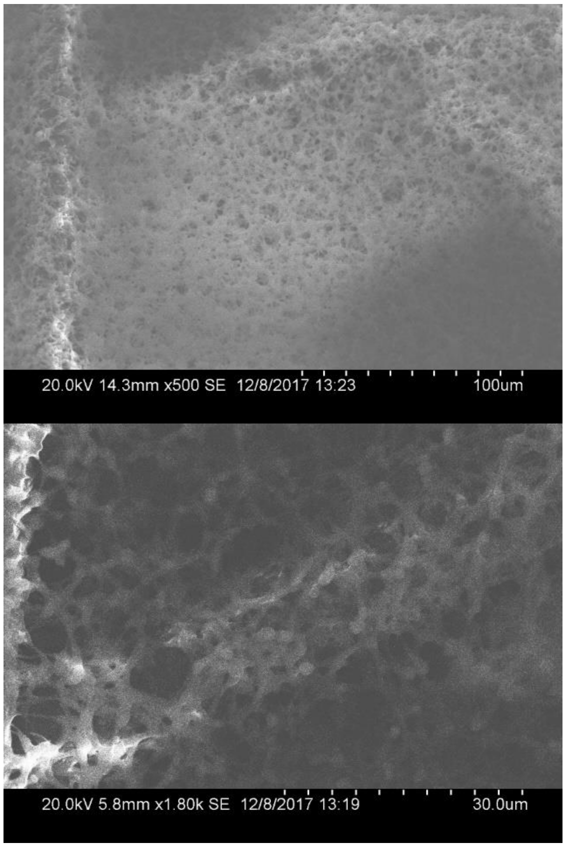

Software Script for Model

```
function [tVec,J,mP,V,C] = expFilt(alpha,vR)
% Matthew Leipner
% Aug 3, 2017

%Inlet Parameters
percA = 50;              %[%]      Percent of beads which will pass through membrane (bead "A")
%vT = 1E-6;              %[m^3]    Total volume to be processed
vT = 1E-3;               %[m^3]    Total volume to be processed
nCalc = 1E2;             %[-]      Number of calculations to complete per cycle (more provides smoother curve)

%Define Constants
%Fluid Flow
%Q0 = 7.57313E-08;       %[m^3/s]  Initial volumetric flow rate (Flux*Area)
Q0 = 1.3678E-5;          %[m^3/s]  Initial volumetric flow rate (Flux*Area)
%Particle Flow
Am = 2.8E-4;             %[m^2]    Effective membrane area
dP = 310E3;              %[Pa]     Driving force (transmembrane pressure drop)
    %***CITE SYRINGE FILTER MANUFACTURER***
Cb0 = 0.5;               %[kg/m^3] Bulk concentration
J0 = Q0*Cb0;             %[kg/s]   Initial particle flow rate = (volume flow rate)*(particle concentration)
mu = 9.0925e-04;         %[Pa*s]   Viscosity of water (analagous to PBS solution)
Rm = dP.*Am./(mu.*J0);   %[m^-1]   Resistance of clean membrane, modified from Darcy's law
    %***CITE TRANSPORT TEXT FOR DARCY'S LAW***

%Constants From Experimental Data
a  = 4.1;                %[m^2/kg] Pore blockage parameter, membrane area blocked/unit mass bead convected to
membrane surface
fR = 2.4E12;             %[m/kg]   Combined f'R', rate of increase of bead layer resistance with time
Rp0 = 4E11;              %[m^-1]   Initial resistance of bead deposit
%        ***CITE HO & ZYDNEY FOR EXPERIMENTAL VALUES USED IN MODEL***

%Calculate time intervals for exp-decay forward, low-resistance forward, and backflush
if alpha == 1
  tVec = 0:(vT./Q0)./nCalc:vT./Q0;   %[s]   Single calculation time vector for alpha of 1
else
  vF = vR.*alpha./(1-alpha);         %[m^3]  Volume pushed forward
  tR = vR./Q0;                       %[s]    Time of backflush and low-resistance forward flush
  tF = vF./Q0;                       %[s]    Time of exp-decay forward flush
  if vF >= vT
    critAlpha = 1/(1+tR*Q0*(tF-tR)/(vT*(tF-tR)));
    %sprintf('inlet alpha of %d is greater than or equal to critical alpha of %d, will calculate as alpha =
1',alpha,critAlpha)
    alpha = 1;                       %Sets function to calculate based on alpha = 1
    tVec = 0:(vT./Q0)./nCalc:vT./Q0;      %[s]   Single calculation time vector for alpha of 1
  else
    rawN = (vT-vF)./(vF-vR);   %[Cycles] Raw number of cycles required to reach desired volume processed
    N = ceil(rawN);            %[Cycles] Rounded number of cylces to be completed in calculations (rounded up)
    fCycle = mod(rawN,1);      %[Cycles] Fraction of forward flush to be completed after final calculations
  end
end
```

```
%Initialize Flux calculations
J = [J0];                %[kg/s]     mass flow rate of particles through membrane vector
mP = [0];                  %[kg]       mass of particles fluxed through membrane vector
V = [0];                 %[m3]       permeate volume vector
C = [Cb0];                 %[kg/m3]    particle concentration vector
if alpha == 1              %Runs single forward flush if alpha = 1
  Cb = Cb0;
  RpVec = (Rm+Rp0).*sqrt(1+(2.*fR.*dP.*Cb.*tVec./(mu.*(Rm+Rp0).^2)))-Rm;
  J  = J0.*exp(-a.*dP.*Cb.*tVec./(mu.*Rm)+(Rm./(Rm+RpVec)).*(1-exp(-a.*dP.*Cb.*tVec./(mu.*Rm))));
  V  = Q0.*tVec;

  %Calculate total permeate particle mass (used for concentration)
  mP = zeros(1,length(tVec));
  for i = 2:length(tVec)
     mP(i) = trapz(tVec(1:i),J(1:i));
  end
  C  = mP./V;
else
%Iterate through number of cycles necessary to process desired volume (appends calculated vectors to total
vectors at each step)
   for i = 1:N

%***********************************************************************************
*******%
      %Forward-Flush Calculations

%***********************************************************************************
*******%
      if i == 1
         Cb = Cb0;
         tVec = 0:tF./nCalc:tF;
         RpVec = (Rm+Rp0).*sqrt(1+(2.*fR.*dP.*Cb.*tVec./(mu.*(Rm+Rp0).^2)))-Rm;
         J  = J0.*exp(-a.*dP.*Cb.*tVec./(mu.*Rm)+(Rm./(Rm+RpVec)).*(1-exp(-a.*dP.*Cb.*tVec./(mu.*Rm))));
%{
         P  = P0.*exp(-a.*DF.*Cb.*tVec./(mu.*Rm)-(a.*exp(-
a.*(mu.*(Rm+Rp0)^2+2.*Cb.*DF.*fR.*tVec)./(2.*fR.*mu.*Rm)).*(Rm+Rp0)...
               .*sqrt((pi./2)+(Cb.*DF.*fR.*pi.*(tVec))./(mu*(Rm+Rp0)^2))...
               .*(-1+erf(sqrt(-a.*(mu.*(Rm+Rp0)^2+2.*Cb.*DF.*fR.*(tVec)).*log(2.71828)./(fR.*mu.*Rm))./sqrt(2))...
               ./(fR.*sqrt(-a.*(mu.*(Rm+Rp0)^2+2.*Cb.*DF.*fR.*(tVec)).*log(2.71828)./(fR.*mu.*Rm)))))...
               -(a.*exp(-a.*(mu.*(Rm+Rp0)^2+2.*Cb.*DF.*fR.*tVec)./(2.*fR.*mu.*Rm)).*(Rm+Rp0)...
               .*sqrt((pi./2))...
               .*(-1+erf(sqrt(-a.*(mu.*(Rm+Rp0)^2).*log(2.71828)./(fR.*mu.*Rm))./sqrt(2))...
               ./(fR.*sqrt(-a.*(mu.*(Rm+Rp0)^2).*log(2.71828)./(fR.*mu.*Rm))))));
%}
         V  = Q0.*tVec;
         %Calculate total permeate particle mass (used for concentration)
         mP = zeros(1,length(tVec));
         for j = 2:length(tVec)
            mP(j) = trapz(tVec(1:j),J(1:j));
         end
         C  = mP./V;

         %Calculate fractional fouling cleared in backflush
```

```
        [~,t2] = size(tVec);                    %Time at which exp-decay forward flush completed
        Rp = (1-alpha).*RpVec(end);             %Changes starting fouling parameter to fraction
    else
        [~,t1] = size(tVec);                    %Time at which low-res forward flush completed
        tCalc  = 0:(tF-tR)./nCalc:(tF-tR);
        RpCalc = (Rm+Rp).*sqrt(1+(2.*fR.*dP.*Cb.*tCalc./(mu.*(Rm+Rp).^2)))-Rm;
        PCalc  = J(end).*exp(-a.*dP.*Cb.*tCalc./(mu.*Rm)+(Rm./(Rm+RpCalc)).*(1-exp(-
a.*dP.*Cb.*tCalc./(mu.*Rm))));
        vCalc  = Q0.*tCalc;
        tVec   = [tVec, tVec(end)+tCalc];
        RpVec     = [RpVec, RpCalc];
        J      = [J, PCalc];
        V      = [V, V(end)+vCalc];

        %Calculate total permeate particle mass (used for concentration)
        mPCalc = zeros(1,length(tCalc));
        for j = 1:length(tCalc)
            mPCalc(j) = trapz(tVec(1:t1+j),J(1:t1+j));
        end
        mP = [mP, mPCalc];
        C  = [C, mPCalc./V(t1+1:end)];

        %Calculate fractional fouling cleared in backflush
        [~,t2] = size(tVec);                    %Time at which exp-decay forward flush completed
        Rp = (1-alpha).*RpVec(end);             %Changes starting fouling parameter to fraction
    end


%*******************************************************************************************
*******%
    %Back-Flush Calculations

%*******************************************************************************************
*******%
    tCalc = 0:tR./nCalc:tR;                    %***MAY NEED TO FIX DISCONTINUITY DUE TO 2 CALCS AT A SINGLE T
HERE***
    J  = [J, zeros(1,length(tCalc))-C(end).*Q0];
    V  = [V, V(end)-Q0.*tCalc];
    mP = [mP,zeros(1,length(tCalc))+mP(end)+J(end).*tCalc];
    C  = [C, zeros(1,length(tCalc))+C(end)];    %Permeate concentration remains const during backflush
    tVec = [tVec, tVec(end)+tCalc];
    RpVec = [RpVec, zeros(1,nCalc+1)+RpVec(end)];


%*******************************************************************************************
*******%
    %Low resistance Forward-Flux Calculations (same volume as backflush period, ignores cake resistance)

%*******************************************************************************************
*******%
    tCalc = 0:tR./nCalc:tR;                    %***MAY NEED TO FIX DISCONTINUITY DUE TO 2 CALCS AT A SINGLE T
HERE***
```

```
        J  = [J, zeros(1,length(tCalc))+C(end).*Q0];    %Subtract P(end) as the value will be negative, resulting in a
positive P
        V  = [V, V(end)+Q0.*tCalc];
        mP = [mP,zeros(1,length(tCalc))+mP(end)+J(end).*tCalc];
        C  = [C, zeros(1,length(tCalc))+C(end)];    %Permeate concentration remains const during backflush
        tVec = [tVec, tVec(end)+tCalc];
        RpVec = [RpVec, zeros(1,nCalc+1)+RpVec(end)];
        PStart = (1-alpha).*(J0-J(end))+J(end);        %Update starting flux based on irr. fouling
    end

%*****************************************************************************************
*******%
    %Compute final fractional cycle

%*****************************************************************************************
*******%
    if fCycle>0
    [~,t3] = size(tVec);                    %Time at which low-res forward flush completed
    tCalc  = 0:fCycle.*(tF-tR)./nCalc:fCycle.*(tF-tR);
    RpCalc = (Rm+Rp).*sqrt(1+(2.*fR.*dP.*Cb.*tCalc./(mu.*(Rm+Rp).^2)))-Rm;
    PCalc  = J(end).*exp(-a.*dP.*Cb.*tCalc./(mu.*Rm)+(Rm./(Rm+RpCalc)).*(1-exp(-a.*dP.*Cb.*tCalc./(mu.*Rm))));
    vCalc  = Q0.*tCalc;
    tVec   = [tVec, tVec(end)+tCalc];
    RpVec     = [RpVec, RpCalc];
    J      = [J, PCalc];
    V      = [V, V(end)+vCalc];

    %Calculate total permeate particle mass (used for concentration)
    mPCalc = zeros(1,length(tCalc));
    for j = 1:length(tCalc)
        mPCalc(j) = trapz(tVec(1:t3+j),J(1:t3+j));
    end
    mP = [mP, mPCalc];
    C  = [C, mPCalc./V(t3+1:end)];
    end
end
C = C./Cb0*100;
%plot(tVec,C)
%Plotting for Paper
%{
figure
hold on
plot(tVec,mP./max(mP),'r-')
plot(tVec,J./max(J),'b')
xlabel('Time [s]')
ylabel('Fraction of maximum [%]')
legend('Permeate solute mass [kg/kg]','Solute Flux [kg/m2s]')
plot(linspace(0,tVec(end)),zeros(1,100)),'k')
axis([0 tVec(end) -1 1])
%}
End
```

```matlab
function plotFilt
% Matthew Leipner
% Nov 7, 2017

%Set constant backflush volume
vR =  1E-4;              %[m^3]      Constant backflush volume

%Plot end concentration vs. Alpha
alpha = 0.55:0.01:1.0;

figure
hold on

%Calculate successive alphas
finalCVec = [];
finalTVec = [];
tVec = {};
CVec = {};
h = waitbar(0,'Initializing waitbar...');
for i = 1:length(alpha)
    waitbar(i./length(alpha),h,sprintf('Currently calculating alpha of %d', alpha(i)))
    [t,~,~,~,C] = expFilt(alpha(i),vR);
    tVec = [tVec, t];
    CVec = [CVec, C];
    finalCVec = [finalCVec, C(end)];
    finalTVec = [finalTVec, t(end)];
    plot(tVec{i},CVec{i},'Color',rand(1,3))
    labeln = num2str(alpha(i));
    %annotation('textarrow',[.1 .2],[.1 .2],'String','my text')
end
waitbar(i./length(alpha),h,'Calculations complete')

axis([0 finalTVec(1).*1.1 0 100])
title('Time vs. Percent Recovery')
xlabel('Time [s]')
ylabel('Percent Recovery [%]')

%Plot alpha vs. final concentration
figure
hold on
plot(linspace(0.5,length(alpha)),zeros(1,100)+83.6740,'r--')
plot(alpha,finalCVec)
axis([0.5 1 0 100])
close(h)
title('Duty Cycle vs. Percent Recovery')
xlabel('Duty Cycle [%]')
ylabel('Percent Recovery [%]')
legend('Maximum Recovery, Alpha -> 0.50+','Recovery with Variable Alpha')

%Plot final time vs. final concentration
figure
hold on
plot(linspace(0,finalTVec(1)),zeros(1,100)+83.6740,'r--')
```

```
plot(finalTVec,finalCVec)
axis([0 finalTVec(1) 0 100])
%title('Process Time vs. Percent Recovery')
xlabel('Process Time [s]','fontsize',24)
ylabel('Percent Recovery [%]','fontsize',24)
legend('Time at Alpha -> 0.50+','Time with Variable Alpha')

%Plot alpha vs. process time
figure
hold on
plot(alpha,finalTVec)
axis([0.5 1 0 finalTVec(1)])
%title('Duty Cycle vs. Process Time')
xlabel('Duty Cycle [%]','fontsize',24)
ylabel('Process Time [s]','fontsize',24)
%legend('Time at Alpha -> 0.50+','Time with Variable Alpha')

%Plot alpha vs. Recovery/Process Time
figure
hold on
plot(alpha,finalCVec./finalTVec)
axis([0.5 1 0 0.5])
title('Duty Cycle vs. Recovery/Process Time')
xlabel('Duty Cycle [%]','fontsize',24)
ylabel('Recovery/Process Time [%/s]','fontsize',24)

% for i = 1:length(alpha)
%     xlswrite('TheoreticalData.xlsx',CVec(i),'Sheet2',sprintf('A%d',i));
% end

end
```

Nomenclature

Symbol	Definition	Units
A_m	effective membrane area	m^2
C_b	bulk solute concentration	kg/m^3
$C_{b,0}$	initial bulk solute concentration	kg/m^3
dz	membrane thickness	M
f	fraction of solute present as aggregates	kg/kg
f'	fraction of solute which contribute to growth of blockage	kg/kg
f'R'	rate of increase of solute layer resistance with time	$m^{-1}s^{-1}$
J	solute flux across membrane	kg/m^2s
k	membrane permeability	kg/m
n	cycle	-
ΔP	transmembrane pressure drop	Pa
Q_0	initial volumetric flow rate	m^3/s
R_m	resistance of clean membrane	m^{-1}
$R_{P,0}$	initial resistance due to solute deposit membrane	m^{-1}
t	time	s
t_p	time at which membrane region is first blocked by solute	s
V	instantaneous volume processed	m^3
V_R	backflush volume	m^3
V_T	total volume to be processed	m^3
α	pore blockage parameter, membrane area blocked/unit mass bead convected to membrane surface	m^2/kg
μ	fluid viscosity	Pa-s
